# Synergistic Chemo-Immunotherapy: Recombinant Fusion Protein-Based Surface Modification of NK Cell for Targeted Cancer Treatment

**DOI:** 10.3390/pharmaceutics16091189

**Published:** 2024-09-08

**Authors:** Su Yeon Lim, Luna Kim, Hongbin Kim, Jeong-Ann Park, Jina Yun, Kwang Suk Lim

**Affiliations:** 1 Department of Smart Health Science and Technology, Kangwon National University, Chuncheon 24341, Republic of Korea; suyeonlim9846@kangwon.ac.kr (S.Y.L.);; 2 Department of Environmental Engineering, College of Art, Culture and Engineering, Kangwon National University, Chuncheon 24341, Republic of Korea; pjaan@kangwon.ac.kr; 3 Division of Hemato-Oncology, Department of Medicine, Soonchunhyang University Bucheon Hospital, Bucheon 14584, Republic of Korea; 19983233@schmc.ac.kr; 4 Department of Biotechnology and Bioengineering, College of Art, Culture and Engineering, Kangwon National University, Chuncheon 24341, Republic of Korea

**Keywords:** cell surface engineering, antibody binding, targeted delivery, antibody–drug conjugates, recombinant fusion protein

## Abstract

While traditional combination anticancer treatments have shown promising results, there remains significant interest in developing innovative methods to enhance and integrate chemotherapy and immunotherapy. This study introduces a recombinant fusion protein-based cell surface modification system that synergistically combines chemotherapy and immunotherapy into a single-targeted chemo-immunotherapy approach. A cell surface-modified protein composed of an antibody-specific binding domain and a cell-penetrating domain rapidly converts immune cells into chemo-immuno therapeutics by binding to antibodies on the surface of immune cells. Utilizing a non-invasive, non-toxic approach free of chemical modifications and binding, our system homogeneously transforms immune cells by transiently introducing targeted cytotoxic drugs into them. The surface-engineered immune cells loaded with antibody–drug conjugates (ADCs) significantly inhibit the growth of target tumors and enhance the targeted elimination of cancer cells. Therefore, NK cells modified by the cell surface-modified protein to incorporate ADCs could be expected to achieve the combined effects of targeted cancer cell recognition, chemotherapy, and immunotherapy, thereby enhancing their therapeutic efficacy against cancer. This strategy allows for the efficient and rapid preparation of advanced chemo-immuno therapeutics to treat various types of cancer and provides significant potential to improve the efficacy of cancer treatment.

## 1. Introduction

In the field of advanced cancer treatment, there is an increasing emphasis on combining chemotherapy and immunotherapy to leverage the synergistic benefits of both modalities and enhance overall clinical anticancer efficacy [1,2]. For chemo-immunotherapy to be effective, it must meet three key criteria: first, chemotherapeutic agents should not only induce cancer cell death but also modulate the immune system to support anticancer immunity; second, chemotherapy should be precisely targeted to minimize adverse effects on immune cells; and third, effector cells of the immune system should maintain their cytolytic capacity against cancer cells [3,4,5]. Developing new approaches that satisfy these conditions could represent a significant advancement in the field of targeted cancer chemo-immunotherapy.

Monoclonal antibodies (mAbs) have become a cornerstone of clinical oncology immunotherapy, serving as effective vehicles for the delivery of cytotoxic agents [6]. Over the past twenty years, the development of antibody–drug conjugates (ADCs) has been remarkable, offering promising clinical results by enhancing the delivery of potent anticancer drugs while reducing off-target side effects [7,8]. Concurrently, significant advances have been made in utilizing immune cells as a novel form of immunotherapy. This progress involves isolating tumor-reactive immune cells from patients, expanding them ex vivo, and then reintroducing them into the patient’s body [9]. A key development in this area is the genetic engineering of immune cells, particularly the creation of chimeric antigen receptor T (CAR-T) cells designed to target specific cancer antigens [9,10]. Although CAR-T cells have garnered considerable clinical attention and support, side effects such as cytokine release syndrome and issues related to the tumorigenesis of administered T cells have been reported [10,11].

Despite the individual successes of ADCs and immune cell therapies, their synergistic combination is now emerging as a promising strategy to enhance clinical anticancer responses [11,12]. In this combinatorial approach, ADCs promote immunogenic cancer cell death, rendering cancer cells more visible and thus easier to target by reintroduced immune cells [13,14]. To realize this therapeutic potential, innovative methods are needed to facilitate the simultaneous delivery of both ADCs and immune cells directly to the tumor site, thereby maximizing their combined efficacy in cancer treatment [15,16].

**Figure 1:**
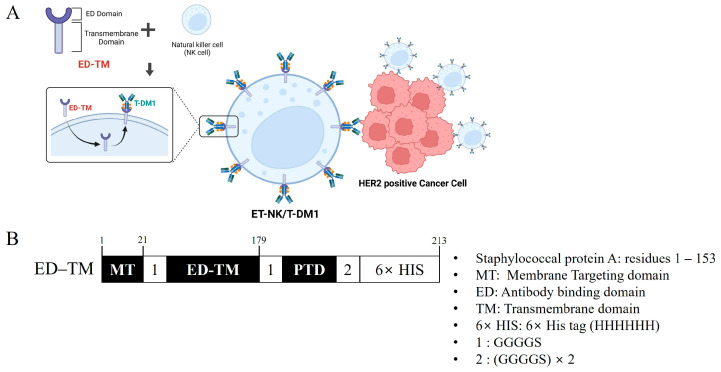
Schematic illustration for targeted chemo-immunotherapy using our cell surface engineering fusion protein. (**A**) The formation process of the ET-NK/T-DM1 complex. (**B**) Structure of the cell surface engineering protein (ED-TM). Structure of ED-TM fusion protein vector constructs. MT: membrane targeting domain; ED: antibody binding domain derived from staphylococcal protein A (residues 1 to 153); TM: transmembrane domain derived from CD8; 1: GGGGS linker; 2: GGGGS × 2 linker; 6× HIS: HHHHHH.

We developed an immune cell surface modification system (ED-TM) that spontaneously transforms immune cells into targeted chemo-immunotherapy agents through the incorporation of ADCs on their surface (Figure 1A). ED-TM, composed of an antibody capture domain and a cell penetration domain (PTD), can be rapidly introduced into immune cells via the PTD and subsequently bind ADCs on the surface through the antibody capture domain. This process leverages the antibody recognition ability of the antibody-specific binding fusion protein introduced into the immune cell. Our immune cell surface modification system allows for the generation of surface-engineered functional immune cells capable of embedding a variety of ADCs without disrupting cell membrane integrity.

The immune cell surface modification system (ED-TM) was designed to modify the surface of NK cells for the incorporation of ADCs. These surface-engineered NK cells (ET-NK), generated using our approach, were incubated with trastuzumab emtansine (T-DM1), a model ADC, to test our hypothesis. ET-NK/T-DM1 cells effectively recognized and destroyed human epidermal growth factor receptor 2 (HER2)-positive cancer cells through the combined effects of T-DM1 and NK cells. This single formulation chemo-immunotherapy, ET-NK/T-DM1 cells, demonstrated higher anticancer efficacy compared to the cotreatment of NK cells and T-DM1. Our novel approach in creating readily available, advanced immune cells for “off-the-shelf” chemo-immunotherapy offers significant therapeutic advantages over traditional methods. This strategy uniquely combines the delivery of antibodies, cytotoxic agents, and immune effector cells directly to the target tumor. This simultaneous application enhances the efficiency and effectiveness of the treatment, providing a more comprehensive and targeted approach to combating cancer cells.

## 2. Materials and Methods

### 2.1. Materials

Trastuzumab emtansine (T-DM1) was obtained from MedChemExpress (Monmouth Junction, NJ, USA). The Cell Counting Kit-8 (CCK-8) was purchased from DOJINDO Laboratories (Kumamoto, Japan). The PE annexin V apoptosis detection kit with 7-AAD was acquired from BioLegend (San Diego, CA, USA). The Human IgG Total Uncoated ELISA Kit was obtained from Invitrogen (Carlsbad, CA, USA). Immobilized Metal Chelate Affinity Chromatography (IMAC) columns, the Trans-Blot Turbo RTA Transfer Kit, Bio-Safe coomassie stain, and Opti-4CN substrate were purchased from Bio-Rad (Hercules, CA, USA). All cancer cell lines, including SK-BR-3, Calu-3, and MDA-MB-231, were acquired from the Korean Cell Line Bank (Seoul, Republic of Korea). Dulbecco’s Modified Eagle’s Medium (DMEM) and RPMI 1640 were obtained from Luscience (Hanam, Republic of Korea). Fetal Bovine Serum (FBS) was purchased from MilliporeSigma (Burlington, MA, USA). Phosphate-Buffered Saline (PBS) and Dulbecco’s Phosphate-Buffered Saline (DPBS) were also purchased from Welgene (Gyeongsan, Republic of Korea). The anti-6× His Tag antibody, anti-rabbit IgG, and HRP-linked antibody were acquired from cell signaling technology (Danvers, MA, USA). All other reagents were of analytical grade. All samples for flow cytometry (FACS Calibur and FACSympony, Becton Dickinson, Franklin Lakes, NJ, USA) and confocal laser scanning microscopy (CLSM; Carl Zeiss, Oberkochen, Baden-Württemberg, Germany) were analyzed in a core facility at Kangwon National University (Chuncheon, Republic of Korea).

### 2.2. Construction and Preparation of the Cell Surface Engineering Protein (ED-TM)

The cell surface engineering protein (ED-TM) consists of two domains: ED and TM. The ED domain is derived from staphylococcal protein A (residues 93–153), and the TM domain is derived from CD8 (cluster of differentiation 8/transmembrane glycoprotein). A 1 kbp DNA fragment encoding the ED and TM domains was sub-cloned into the pET-28a expression vector by COSMOgenetech (Seoul, Republic of Korea). The ED-TM recombinant fusion protein was transformed into *Escherichia coli* strain BL21 (*E. coli* BL21) and cultured in LB medium with 100 µg/mL kanamycin at 37 °C for 4 h until the OD at 600 nm reached 0.4–0.6. One millimole of IPTG (isopropyl β-D-1-thiogalactopyranoside) was added to induce protein expression, and then the cells were cultured for 16–18 h at 20 °C and 120 rpm. After induction, cell pellets were collected by centrifugation and subjected to sonication for 8 cycles of 20 s each. The ED-TM recombinant fusion protein was purified using fast protein liquid chromatography (FPLC) equipped with a Ni^2+^-NTA column (NGC, Bio-Rad, Hercules, CA, USA). The molecular weight of the ED-TM protein after dialysis was determined by SDS-PAGE. Identification of ED-TM was confirmed by Western blot assay using an anti-6× His antibody (1:1000).

### 2.3. Cell Culture

HER2-positive cancer cells such as SK-BR-3 and Calu-3 were maintained according to the protocols provided by the Korean Cell Line Bank (KCLB, Seoul, Republic of Korea). MDA-MB-231, a HER2-negative cancer cell line, was also cultured according to KCLB’s protocols. SK-BR-3 and MDA-MB-231 human breast cancer cells were cultured in RPMI 1640 medium with 10% fetal bovine serum (FBS) and supplemented with 100 U/mL penicillin and 100 µg/mL streptomycin. Calu-3 lung cancer cells were maintained in DMEM containing the same supplements as mentioned above. All cancer cells were incubated at 37 °C and 5% CO_2_ under humidified conditions and subcultured every 2 or 3 days depending on their confluency. NK-92 cells were purchased from ATCC (American Type Culture Collection, Manassas, VA, USA) and cultured according to ATCC’s protocols. NK-92 cells were cultured in MEM alpha medium containing 20% FBS, 1% penicillin/streptomycin (P/S), 4 mM folic acid, 20 mM inositol, 0.02 mM 2-mercaptoethanol and 100 unit/mL interleukin-2 (IL-2). NK-92 cells were incubated at 37 °C and 5% CO_2_ under humidified conditions and subcultured every 2 days depending on their confluency.

### 2.4. Cytotoxicity Test of ED-TM Fusion Protein in NK Cell

To determine the cytotoxicity of the ED-TM fusion protein in NK cells, NK cells were treated with ED-TM fusion protein from 0 µg to 20 µg for 24 h at 37 °C and 5% CO_2_. NK cell viability was measured by CCK-8 assay according to the manufacturer’s protocol. The optical density in the well plate was measured by a microplate reader (BioTek, Winooski, VT, USA) at 450 nm.

### 2.5. Optimization of Modification Condition to Generate ET-NK Cell Using ED-TM

Rhodamine and fluorescein (FITC) were conjugated with ED-TM and trastuzumab emtansine (T-DM1), respectively, to optimize the modification conditions of NK cells using the ED-TM fusion protein. Rhodamine-conjugated ED-TM was added to NK cells at varying concentrations of ED-TM and incubated at 37 °C and 5% CO_2_ for 5 h and 12 h. Surface-engineered NK cells with ED-TM (ET-NK) were centrifuged at 1300 rpm for 5 min at room temperature (RT) to remove the unbound ED-TM fusion protein. T-DM1 at 20 µg was added to ET-NK cells for 15 min at RT. ET-NK/T-DM1 cells were also centrifuged at 1300 rpm for 3 min and washed twice with DPBS to eliminate unbound T-DM1. All test groups were detached from the well plate and then fixed with 4% paraformaldehyde in 1× PBS. All samples were kept in a refrigerator at 4 °C until CLSM analysis. Fluorescence in NK cells with rhodamine-conjugated ED-TM and FITC-T-DM1 was observed using CLSM in a core facility at Kangwon National University (Chuncheon, Republic of Korea).

### 2.6. Confirmation of the Formation of ET-NK/T-DM1 Using Optimization Condition

To test whether ED-TM and T-DM1 were located on the surface of NK cells, we prepared rhodamine- and FITC-conjugated ED-TM and T-DM1, respectively. Rhodamine-conjugated ED-TM was added to NK cells for 12 h and then mixed with FITC-conjugated T-DM1 for 15 min. After incubation, ET-NK/T-DM1 cells were prepared for CLSM analysis according to the following procedure. ET-NK/T-DM1 cells were washed with DPBS three times and then treated with 4% paraformaldehyde in 1× PBS and kept in a refrigerator at 4 °C until CLSM and FACS analysis. The fluorescence of rhodamine and FITC was analyzed with CLSM and FACS in a core facility at Kangwon National University (Chuncheon, Republic of Korea).

### 2.7. In Vitro Anticancer Activity

To determine the anticancer efficacy of ET-NK/T-DM1, HER2-positive cancer cells including SK-BR-3 and Calu-3 and HER2-negative cancer cell such as MDA-MB-231 were labeled with 20 μM Cell Tracker blue CMAC. All cancer cells were seeded at a population of 1 × 10^5^ cells/well on a 24-well plate and then incubated for 24 h at 37 °C and 5% CO_2_. Cancer cells were co-incubated with T-DM1, NK cells, and the NK + T-DM1 cotreatment at an effector-to-target (*E:T*) ratio of 1:1. ET-NK/T-DM1 was also added to the cancer cells at an *E:T* ratio of 1:1 and 10:1, respectively. After 2 h, all treatments were washed twice with DPBS and then the remaining cancer-bound effector cells were further incubated for 24 h. Cancer cell viability was confirmed by an annexin V apoptosis detection kit according to the manufacturer’s protocol and analyzed by FACS in a core facility at the Kangwon National University (Chuncheon, Republic of Korea).

### 2.8. Statistical Analysis

All data are represented as the mean ± standard deviation (*n* = 3). Statistical analysis was performed with Student’s *t*-test. Values of * *p* < 0.05, ^#^ *p* < 0.01, and ^##^ *p* < 0.001 were considered statistically significant.

## 3. Results

### 3.1. Schematic Illustration Showing the Plasmid Constructs of the Cell Surface Engineering Protein

The cell surface engineering fusion protein (ED-TM) consists of an ED domain derived from staphylococcal protein A (residues 1–153) and a TM domain derived from the transmembrane domain of CD8 (Figure 1B). ED-TM is composed of a sequence similar to that of the existing CAR system and could be located on the cell membrane of immune cells after intracellular uptake. In particular, the cancer cell recognition site of the existing CAR system was replaced with an ED domain that could bind to antibodies, enabling binding to various antibodies. The sequence of ED-TM was inserted into the pET-28a protein expression vector to generate the cell surface engineering fusion protein (ED-TM). The Tat domain in the ED-TM fusion protein is a well-known protein transduction domain (PTD) that enhances the cellular uptake of the fusion protein. After intracellular uptake by the Tat domain, the ED-TM fusion protein is transported to the cell membrane by the membrane targeting domain (MT), resulting in the cell surface-modified NK cell (ET-NK).

### 3.2. Characterization and Cytotoxicity Test of ED-TM Protein

**Figure 2:**
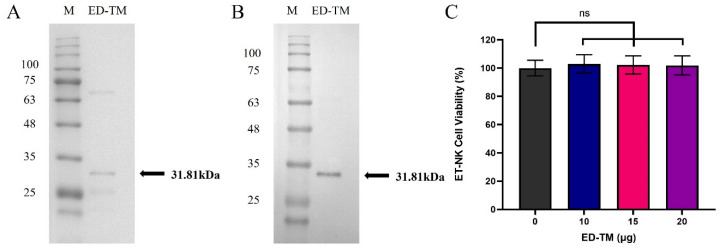
Characterization and cytotoxicity test of ED-TM fusion protein. (**A**) Verification of the ED-TM fusion protein by SDS-PAGE after purification. (**B**) Identification of the ED-TM fusion protein by Western blot using an anti-6× his antibody (1:1000 dilution). (**C**) Comparison of NK cell viability between unmodified NK cell (0 μg) and ET-NK cells generated with 10, 15 or 20 μg of ED-TM fusion protein for 12 h. The cytotoxicity test of the ED-TM fusion protein in NK cells was performed using Cell Counting Kit-8 (CCK-8). M: Marker; ED-TM: Cell surface engineering fusion protein; ns: not significant.

The ED-TM fusion protein, after purification, was verified by SDS-PAGE and Western blot. As shown in Figure 2A,B, ED-TM revealed a band upon the Western blot analysis with an anti-6× His antibody near the expected size range at 31.81 kDa. The ED-TM fusion protein at concentrations of 10, 15 or 20 μg was added to NK cells for the confirmation of cytotoxicity and then incubated for 12 h. The cytotoxicity of all treatment groups in NK cells was measured by CCK-8 assay. As shown in Figure 2C, the viability of the NK cell was approximately 100% in all treated groups. The ED-TM fusion protein did not induce any cytotoxicity in NK cells at the treatment concentrations used. Based on this experiment, we prepared the cell surface-modified NK cell by ED-TM (ET-NK) using 20 μg of the ED-TM fusion protein.

### 3.3. Optimization of Cell Surface Modification Condition Using ED-TM Protein

**Figure 3:**
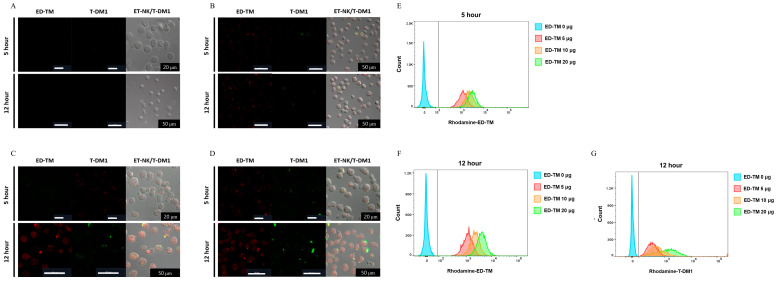
Optimization of modification condition to generate ET-NK cells using ED-TM. To generate ET-NK cells using the ED-TM fusion protein, various amounts of ED-TM were added to NK cells and then incubated for 5 or 12 h. ED-TM was conjugated with rhodamine. FITC-conjugated T-DM1 and rhodamine-conjugated T-DM1 were prepared. (**A**) 0 μg of ED-TM, (**B**) 10 μg of ED-TM, (**C**) 15 μg of ED-TM, (**D**) 20 μg of ED-TM, (**E**) NK cells with ED-TM for 5 h, (**F**) NK cells with ED-TM for 12 h. T-DM1 at 20 μg was added to the ET-NK cell for 15 min to incorporate T-DM1 on the NK cell surface modified by ED-TM. Fluorescence on the ET-NK/T-DM1 was observed using CLSM and FACS. (**G**) Rhodamine-conjugated T-DM1 was incubated for 15 min with ET-NK that was prepared using different concentration of ED-TM protein for 12 h. Fluorescence of T-DM1 on ET-NK/T-DM1 was detected using FACS. ED-TM: Cell surface engineering fusion protein; T-DM1: trastuzumab emtansine; ET-NK: NK cell modified by ED-TM; ET-NK/T-DM1: ET-NK cell incorporated with T-DM1; FITC: fluorescein; CLSM: confocal laser scanning microscopy; FACS: fluorescence-activated cell sorting.

To generate cell surface-modified NK cells (ET-NK) using the ED-TM fusion protein, various amounts of ED-TM fusion protein were added to NK cells and then incubated for 5 or 12 h. As shown in Figure 3, rhodamine-conjugated ED-TM was located in the membrane of NK cells after incubation for 5 and 12 h. FITC-conjugated T-DM1 was also observed on the surface of NK cells with ED-TM in an ED-TM concentration-dependent manner. ED-TM sufficiently modified 100% of NK cells after 5 h, while the incorporation of T-DM1 into ET-NK was lower compared to incubation for 12 h. T-DM1 was not detected in NK cells without ED-TM in Figure 3. The ET-NK cell was prepared using different concentrations of ED-TM protein for 12 h to confirm incorporation of T-DM1 by ED-TM on the surface of the ED-NK cell. As shown in Figure 3G, rhodamine-conjugated T-DM1 was increased on the ET-NK cell depending on the concentration of the ED-TM protein. These results suggest that the ED-TM can capture T-DM1 on the surface of NK cells.

### 3.4. Confirmation of the Formation of ET-NK/T-DM1

**Figure 4:**
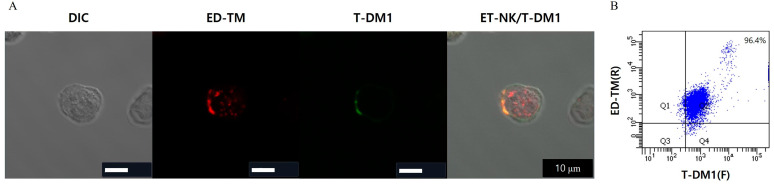
Confirmation of the formation of ET-NK/T-DM1 using optimized condition. (**A**) CLSM image of ET-NK/T-DM1. (**B**) FACS analysis of ET-NK/T-DM1. ED-TM at 20 μg was added to NK cells to prepare ET-NK cells for 12 h at 37 °C and 5% CO_2_. The ET-NK cells were mixed with T-DM1 at 20 μg for 15 min at room temperature, resulting in the formation of ET-NK/T-DM1. ED-TM and T-DM1 were conjugated with rhodamine and FITC, respectively. Fluorescence on the ET-NK/T-DM1 was observed using CLSM. ED-TM: cell surface engineering fusion protein; T-DM1: trastuzumab emtansine; ET-NK: NK cell modified by ED-TM; ET-NK/T-DM1: ET-NK cell incorporated with T-DM1; FITC: fluorescein; CLSM: confocal laser scanning microscopy.

To confirm the formation of ET-NK/T-DM1 under optimized conditions, we observed the location of ED-TM and T-DM1 on the surface of NK cells. NK cells were incubated with 20 μg of ED-TM for 12 h, and then T-DM1 was mixed with ET-NK cells for 15 min at room temperature. In Figure 4A, rhodamine-conjugated ED-TM and FITC-conjugated T-DM1 were observed on the surface of NK cells. FITC-conjugated T-DM1 was only detected in the same location as rhodamine-conjugated ED-TM on the surface of NK cells. Almost 100% of the cells were dual-positive for rhodamine and FITC, indicating that the entire population of NK cells was transformed into ET-NK/T-DM1, as shown in Figure 4B. This result indicates that the ED domain in the ED-TM fusion protein can capture T-DM1 on the surface of NK cells.

### 3.5. In Vitro Anticancer Efficacy of ET-NK/T-DM1

**Figure 5:**
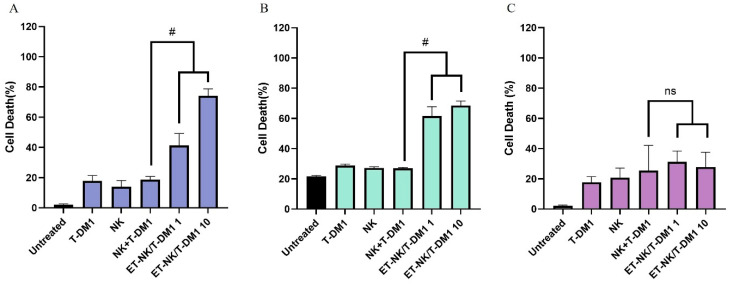
In vitro anticancer effect of ET-NK/T-DM1. To confirm the anticancer effect of ET-NK/T-DM1, SK-BR-3, Calu-3 and MDA-MB-231 cell were labeled with Cell Tracker blue CMAC. (**A**) SK-BR-3; (**B**) Calu-3; (**C**) MDA-MB-231. All cancer cell were co-incubated with NK cells, T-DM1, NK cell + T-DM1 co-treatment and ET-NK/T-DM1 for 2 h and then washed with DPBS two times. NK and NK + T-DM1 co-treatment were added to the cancer cell at an *E:T* ratio of 1:1. ET-NK/T-DM1 was added to the cancer cell at an *E:T* ratio of 1:1 and 10:1, respectively. They were further incubated for 24 h and then prepared to measure cancer cell death using the annexin V apoptosis detection kit. Cancer cell death was analyzed by FACS at the core facility of the Kangwon National University. ED-TM: cell surface engineering fusion protein; T-DM1: trastuzumab emtansine; ET-NK: NK cell modified by ED-TM; ET-NK/T-DM1: ET-NK cell incorporated with T-DM1; FACS: flow cytometry. Data represent mean ± standard deviation (ns, not significant, ^#^ *p* < 0.01).

We determined the anticancer efficacy of the ET-NK/T-DM1 in HER2-positive cancer cells including SK-BR-3 and Calu-3 and HER2-negative cancer cells such as MDA-MB-231 cells. To validate the therapeutic advantages of ET-NK/T-DM1 over the NK + T-DM1 cotreatment and NK alone, each of the cell lines was incubated with T-DM1 and NK cells and the NK + T-DM1 cotreatment at an effector-to-target (*E:T*) ratio of 1:1 for 2 h. ET-NK/T-DM1 was co-incubated with each cancer cell at an E:T ratio of 1:1 and 10:1 for 2 h, respectively. After incubation, all cells were washed with PBS two times. Subsequently, the cancer cells were further incubated for 24 h, and the resulting cancer cell death was measured. As shown in Figure 5, ET-NK/T-DM1 significantly improved the cancer cell death in HER2-positive cell lines such as SK-BR-3 and Calu-3 compared with other treatment groups. ET-NK/T-DM1 showed enhancement of the anticancer effect in SK-BR-3 depending on the E:T ratio, as shown in Figure 5A. In addition, they showed sufficient anticancer effects even at low concentrations in Calu-3, as shown in in Figure 5B. ET-NK/T-DM1 exhibited enhanced anticancer efficacy in HER2-positive cancers due to the HER2-targeting effects of T-DM1 introduced by ED-TM, while no significant cell death was observed in HER2-negative MDA-MB-231 cells.

## 4. Discussion

Chemo-immunotherapy has achieved groundbreaking advancements in oncology by synergistically utilizing the direct cytotoxic effects of chemotherapy and the precise targeting ability of immunotherapy [3,17]. This approach takes advantage of the best of both worlds by leveraging the immune system’s ability to selectively target cancer cells while deploying chemotherapy agents to more effectively eliminate these cells [17,18]. Importantly, chemo-immunotherapy offers improved efficacy while potentially reducing systemic toxicity, paving the way for more effective and patient-friendly cancer treatment [19,20]. Despite progressive advances in chemo-immunotherapy, existing development methodologies face practical limitations [21,22]. These include the challenges of effectively fusing antibody–drug conjugates (ADCs) with immune cell therapeutics to promote synergistic tumor responses within the tumor microenvironment. There are also complex issues associated with simultaneously delivering ADCs and genetically modified immune cells, such as chimeric antigen receptor T (CAR-T) cells, to the tumor site and ensuring optimal interaction [15,23,24]. Additionally, the customized and complex nature of these therapeutic strategies poses significant production and storage challenges, limiting their widespread clinical application.

In this study, we developed a novel immune cell surface modification system (ED-TM) that couples antibody–drug conjugates (ADCs) to the immune cell surface to convert immune cells into targeted chemo-immuno therapeutics. In our previous work, we reported a one-step method to directly conjugate DMPE-PEG to T-DM1, which can be introduced by hydrophobic bonding [15]. By chemically conjugating the polymeric lipid to the antibody, we were able to rapidly introduce it to the surface of immune cells and confirm its anticancer efficacy with excellent targeting ability. Once the antibody–drug conjugate was stably introduced to the surface of immune cells, it was demonstrated that the targeting ability of ADCs and cancer cell killing by immune cells, in combination with chemotherapy, were possible. Since previous studies required the chemical binding of antibodies to polymeric lipids, we developed the ED-TM system to introduce ADCs to immune cells without chemical modification in the antibody or ADC.

The immune cell-modified engineered fusion protein system (ED-TM) was composed of an ED domain for antibody capture, a TM domain for cell membrane introduction, and a Tat sequence, which is a cell-penetrating domain. Upon treatment of NK cells with the ED-TM protein, ED-TM was rapidly introduced into the cell by the Tat sequence and positioned at the cell membrane by the membrane-targeting domain (MT) and transmembrane domain of CD8 (TM) (Figure 3). After ED-TM was translocated to the cell membrane by MT, the ED domain was exposed on the cell surface to capture antibodies and allow them to be introduced to the surface of the immune cell. As shown in Figure 3 and Figure 4, T-DM1 was identified at the same site on the cell membrane where ED-TM was located. Notably, in NK cells (ET-NK) whose surface was modified by ED-TM, T-DM1 was introduced within a reaction time of 15 min. The rapid introduction time of T-DM1 was not significantly different from the polymeric lipid system used in previous studies [15]. The spontaneous binding between the protein and antibody, without chemical modification of T-DM1, is sufficient to introduce T-DM1 to the surface of immune cells. The binding affinity of ED-TM and protein A to antibodies was 4.25 nm and 1.35 nm, respectively. ED-TM was derived from two of the five antibody binding site domains of protein A, so its binding affinity was determined to be low compared to protein A. The fact that the existing antibody could be applied as is suggests that various antibodies or ADCs can be introduced to the cell surface of ET-NK cells in the future. Additionally, it might be possible to utilize this system as a targeted delivery mechanism for stem cells and adult cells, in addition to immune cells. To optimize the incorporation speed and efficiency of ED-TM on the cell surface for its application in various targeted cell delivery systems, further research into optimization strategies is essential.

ET-NK cells (ET-NK/T-DM1), into which T-DM1 (trastuzumab emtansine) has been introduced, were tested for their anticancer effects in the HER2-positive cancer cell lines SK-BR-3 and Calu-3. Compared to the simultaneous T-DM1 treatment group, ET-NK/T-DM1 showed excellent anticancer effects (Figure 5A,B). However, in the HER2-negative cancer cell line MDA-MB-231, there was no significant difference among all treatment groups. The trastuzumab component of T-DM1 introduced to the surface of NK cells by ED-TM demonstrates that it can recognize HER2-positive cancer cells and deliver NK cells to these targets. This result indicates that T-DM1 can be effectively introduced to the surface of cells by the ED-TM protein while maintaining its function.

Surface engineering of natural killer (NK) cells with trastuzumab emtansine (T-DM1) has demonstrated a capacity for concurrent accumulation of both T-DM1 and NK cells within targeted tumor tissues. T-DM1 binding to HER2-positive cancer cells via engineered NK cells disrupts critical cell signaling and microtubule networks, inducing apoptosis. This engineering significantly enhances NK cell migration and cytolytic activity against cancer cells. In proximity to these cancer cells, ET-NK/T-DM1 cells recognize damage-associated molecular patterns (DAMPs) on apoptotic cells, effectively leveraging their innate cytotoxic potential in a targeted therapeutic approach.

While cancer immune cell therapies such as NK cells and T cells demonstrate remarkable anticancer efficacy, several challenges must be addressed before they can be widely applied as treatments. These challenges include cytokine release syndrome (CRS), off-target cell damage, and the risk of autoimmunity. Enhancing the targeting capabilities of these therapies may offer a solution to mitigate these issues. The recombinant fusion protein-based cell surface modification system (ED-TM) can significantly improve allogeneic immune cell therapy by providing a solution with high tumor-specific cytotoxicity and low side effects through non-chemical modification. This approach overcomes the limitations of existing CAR-T cell therapies, such as viral vectors, long production times and limited solid tumor efficacy, by rapidly and cost effectively generating tumor-targeting immune cells at the bedside. This method synergizes antibodies, chemotherapy agents and immune cells to enhance efficacy in solid tumors. Additionally, various previously approved antibodies and antibody–drug conjugates can be applied to immune cell treatments to develop customized therapies. This versatility presents an efficient production method for generating powerful tumor-reactive immune cells in cancer treatment.

## 5. Conclusions

Our study established a recombinant fusion protein-based immune cell surface modification platform for generating advanced forms of chemo-immunotherapy. By introducing an antibody–drug conjugate to the surface of NK cells, we demonstrated excellent anticancer efficacy along with the ability to target solid tumors, highlighting its potential as a powerful treatment method. Our approach facilitates the introduction of a variety of antibody–drug conjugates and antibodies onto the surface of immune cells. This versatility can be expanded to a variety of immune cells, making it a powerful tool against a wide range of cancers. Considering the continuous development of new antibody-based drugs and allogeneic immune cells, our platform’s ability to apply existing antibodies and antibody–drug conjugates without chemical modification significantly increases its applicability. Our approach is designed to be modular, allowing for customized combinations of allogeneic immune cells and specific ADCs to meet the needs of patient-specific therapeutics. In the future, we anticipate that our method will pave the way for a wide range of targeted chemo-immunotherapy options, with the goal of creating readily available “off-the-shelf” therapeutic reagents.

## Data Availability

The original contributions presented in the study are included in the article, further inquiries can be directed to the corresponding author.
